# A novel formulation of Mtb72F DNA vaccine for immunization against tuberculosis

**DOI:** 10.22038/ijbms.2020.41806.9881

**Published:** 2020-06

**Authors:** Razieh Dalirfardouei, Mohsen Tafaghodi, Zahra Meshkat, Adel Najafi, Aida Gholoobi, Maryam Sadat Nabavinia, Samineh Sajedifar, Mojtaba Meshkat, Ali Badiee, Mohammad Ramezani, Abdol-Reza Varasteh, Mahboubeh Naderinasab

**Affiliations:** 1 Research Center for Molecular Medicine, Hamadan University of Medical Sciences, Hamadan, Iran; 2 Department of Medical Biotechnology, Faculty of Medicine, Mashhad University of Medical Sciences, Mashhad, Iran; 3 Nanotechnology Research Center, Institute of Pharmaceutical Technology, Mashhad University of Medical Sciences, Mashhad, Iran; 4 Antimicrobial Resistance Research Center, Mashhad University of Medical Sciences, Mashhad, Iran; 5 Laboratory Division, Fatemieh Hospital, Hamadan University of Medical Sciences, Hamadan, Iran; 6 Medical Genetics Research Center, Mashhad University of Medical Sciences, Mashhad, Iran; 7 Department of Pharmacognosy, Faculty of Pharmacy and Pharmaceutical Sciences Research Center, Shahid Sadoughi University of Medical Sciences, Yazd, Iran; 8 Mashhad University of Medical Sciences, Mashhad, Iran; 9 Mashhad Branch, Isalmic Azad University, Mashhad, Iran; 10 Immunobiochemistry Lab, Allergy Research Center, Mashhad University of Medical Sciences, Mashhad, Iran

**Keywords:** CpG ODN, DNA vaccine, Mtb72F, Mycobacterium tuberculosis, PLGA nanoparticles, Tuberculosis vaccine

## Abstract

**Objective(s):**

*Mycobacterium tuberculosis* (*M. tuberculosis*), an intracellular pathogen, causes 1.5 million deaths globally. Bacilli Calmette-Guérin (BCG) is commonly administered to protect people against *M. tuberculosis* infection; however, there are some obstacles with this first-generation vaccine. DNA vaccines, the third generation vaccines, can induce cellular immune responses for tuberculosis (TB) protection. In this study, optimized DNA vaccine (pcDNA3.1-Mtb72F) entrapped in poly (lactic-co-glycolic acid) (PLGA) nanoparticles (NPs) was used to achieve higher immunogenicity.

**Materials and Methods:**

Plasmid Mtb72F was formulated in PLGA NPs using double emulsion method in the presence of TB10.4 and/or CpG as an adjuvant. Female BALB/c mice were immunized either with NP-encapsulated Mtb72F or naked Mtb72F with or without each adjuvant, using the BCG-prime DNA boost regimen.

**Results:**

These NPs were approximately 250 nm in diameter and the nucleic acid and protein encapsulation efficiency were 80% and 25%, respectively. The NPs smaller than 200 nm are able to promote cellular rather than humoral responses. The immunization with the formulation consisting of Mtb72F DNA vaccine and TB10.4 entrapped in PLGA NPs showed significant immunogenicity and induced predominantly interferon-ɣ (IFN-ɣ) production and higher INF-ɣ/interleukin-4 (IL-4) ratio in the cultured spleen cells supernatant.

**Conclusion:**

PLGA NPs loaded with Mtb72F DNA-based vaccine with TB10.4 could be considered as a promising candidate for vaccination against TB. These results represent an excellent initial step toward development of novel vaccine for TB protection.

## Introduction

Traditional vaccines either consisting of killed pathogens or live attenuated ones drastically decreased the incidence of widespread infectious diseases in the globe in the last century. These vaccines could not effectively prevent some diseases including tuberculosis (TB) and human immunodeficiency viruses (HIV). It is necessary to extend the investigation for alternative vaccine (1). 

*Mycobacterium tuberculosis *(*M. tuberculosis*) is an intracellular pathogen, which causes 1.5 million deaths per year in the world. In 2016, world health organization (WHO) reported the disease as ninth cause of death worldwide. Bacilli Calmette-Guérin (BCG) is a live attenuated vaccine, which is widely administered for protection against *M. tuberculosis *since 1945*.* It is relatively safe and inexpensive stimulating both cellular and humoral immune responses. However, the protective efficacy of BCG is extremely hampered by increasing inconsistent efficacy against the pulmonary disease among different populations, increasing prevalence of chemoresistant TB, and the increased susceptibility of the HIV-infected population (2). It is of great interest to develop a safe and more effective vaccine to show the ability to boost BCG-primed immune responses or even to replace BCG. This type of vaccine can be used in patients with immunodeficiency diseases like HIV-infected individuals, and may have a potential for treating drug resistant TB, which is a top global priority in innovative research (3, 4). Several *M. tuberculosis* antigens were characterized to elicit T-cell and antibody responses. It is well-established that Mtb72F, a fusion protein of Mtb39A (Rv0125) encoding PepA and Mtb32A (Rv1196) encoding PPE18 (5), provide enough safety and efficacy on latent *M. tuberculosis* infection cases. The results of phase 2b controlled trial showed 54.0% protection against active pulmonary TB infection (6). In addition, TB10.4 encoded by the Rv0288 gene belongs to Esat-6 family, which is important for the virulence of tuberculosis (7). TB10.4 stimulates Th1 CD4^+^ immune cell responses and induces protective response against *M. tuberculosis *(8)*.* We have previously constructed recombinant Mtb72F plasmid as a DNA vaccine and produced TB10.4 recombinant protein as an immunogenic antigen and/or adjuvant (9, 10).

DNA vaccines are classified as third-generation vaccines stimulating both cellular and humoral immune system, which make them suitable for prevention and therapy of bacterial and viral pathogens (11, 12). The key issue for DNA vaccination is insufficient transfer of DNA into the antigen presenting cells (APCs) followed by inadequate antigen expression (11). To enhance the uptake of nucleic acids by the cell, different methods including electroporation, live bacteria, and polymeric micro- and nanoparticles are employed (13-15). 

Poly (lactic-co-glycolic acid) (PLGA) polymer approved by the food and drug administration (FDA) for human usage are widely used for the drug delivery, vaccine, and tissue engineering (16, 17). Encapsulation of DNA vaccine in PLGA nanoparticles (NPs) is an attractive antigen delivery system due to its preventing DNA degradation, mimicking pathogens, enhancing internalization by APCs, and long-term antigen release (18, 19). Taken together, we sought to investigate immune response against PLGA-encapsulated Mtb72F DNA vaccine in the presence of either TB10.4 recombinant protein or CpG oligonucleotide as an adjuvant in the animal model. Additionally, we aimed to elicit the immune responses against DNA vaccines in a BCG prime-boost regimen.

## Materials and Methods

***Polymers and reagents***

**Poly (DL-lactide-co-glycolide) (L:G= 75:25, M 50 kDa, inherent viscosity 0.88 dl/g) was purchased from Sigma Aldrich, Chemie Gmbh, Munich, Germany.** Polyvinyl alcohol (PVA) (MW 30–80 kDa, 88% hydrolyzed) was from Polysciences Inc., PA. CpG was obtained from Microsynth, Swiss. *Mycobacterium bovis* BCG (Intravesical BCG) was purchased from Pasteur institute of Iran. 

***Preparation of pcDNA3.1/Mtb72F vaccine and TB10.4 recombinant protein***

**A plasmid pcDNA3.1 containing** *mtb32C, mtb39*, and *mtb32N *was prepared to produce Mtb72F recombinant construction as described previously (9). In our previous study, expression of the recombinant Mtb72F inserted into the pcDNA3.1 was confirmed in CHO mammalian cell line (9). The recombinant pcDNA3.1 was then transformed into *Escherichia coli* DH 5a cells and prepared on a large scale for isolation of pure plasmid DNA. The pcDNA3.1/Mtb72F was subsequently purified using Genopure Plasmid Maxi kit (Roche, Germany). The concentration and purity of pcDNA3.1/Mtb72F was measured by spectrophotometer (NanoDrop^TM^ 2000, Thermo Fischer Scientific). TB10.4 recombinant protein was kindly gifted by Dr Varasteh (10).

***Preparation of PLGA nanoparticles***

Water-oil-water (W/O/W) double emulsion method was used to prepare biodegradable PLGA NPs. The method is essentially the same as that used by Mohaghegh and Tafaghodi (20) with some modifications. NPs loaded with pcDNA3.1/Mtb72F vaccine, and adjuvants including TB10.4 and CpG. Briefly, 200 µl of aqueous solution including pcDNA3.1/Mtb72F (100 µl, 2.4 mg/ml) and either TB10.4 (40 µl, 0.5 mg/ml) or CpG (60 µl, 0.6 mg/ml) was added into PLGA solution (1 ml, 4% w/v) in dichloromethane (DCM, Merck Millipore®, Germany), and sonicated for 30 sec at 80% amplitude on ice bath to form the primary emulsion. 

The resulting W/O emulsion was immediately poured into a 2.5 ml of 1% (w/v) PVA and sonicated twice for 30 sec at 80% amplitude on ice bath and 1 min interval to produce W1/O/W2 emulsion (adding 20 mg of span-80 as emulsifier). Finally, the W1/O/W2 emulsion was poured into a glass beaker containing 25 ml of 0.5% PVA and stirred for approximately 3 hr at room temperature to evaporate DCM. The NPs were isolated by centrifugation at 14000 rpm for 15 min at 4 ˚C. The NPs pellet was washed with deionized water three times and then freeze-dried. The final product was stored at -20 ^°^C. 

***Nanoparticle characterization***

**Size distribution analysis**

Characteristics of DNA vaccine-loaded NPs were evaluated by assaying their diameter, encapsulation efficiency (EE), and stability. The diameter and size distribution of NPs was examined by dynamic light scattering (Malvern Instrument Ltd, UK). 

**Encapsulation efficiency of DNA**

In order to evaluate the EE of pcDNA3.1/Mtb72F and CpG, the amount of unloaded nucleic acid was measured in accordance with previous study (21). After the first centrifugation for NPs isolation, the clear supernatant was collected and the amount of DNA was measured by modified fluorimetric method (2). This method was easily customized for use with SYBR Gold and the bacterial DNA for standard curve. Concentrations of nucleic acid standard solutions were determined by standard ultraviolet absorption spectroscopy. DNA-containing samples were mixed with SYBR Gold solution (Thermo Fischer Scientific) in 1× final concentration, and fluorescence signal was measured within 30 min at 480 nm excitation and 520 nm emission at RT using a Fluorometer (PerkinElmer). Nucleic acid EE% was calculated using the following equation: 

EE%=(total DNA-free DNA)/(total DNA)×100

**Encapsulation efficiency of protein**

In an attempt to assess protein EE, the method explained by De Temmerman *et al.* was used (22). Briefly, the clear supernatant containing non-encapsulated protein was collected after NPs synthesis, and the protein concentration was measured by Bradford assay (Bio-Rad). Protein EE% was calculated according to the following equation:

EE%=(total protein-free protein)/(total protein)×100

***Animals***

Male BALB/c mice at the age of 7-8 weeks were purchased from Pasteur Institute, Tehran. The animals were kept at 12:12 hr light-dark cycle with free access to food and water. All animal experiments were approved by and performed according to the Ethics Committee of Mashhad University of Medical Sciences. The experiments conformed to the international guidelines (23) for the ethical use and care of animals. 

***Immunization procedures***

In order to investigate the function of pcDNA3.1/Mtb72F -encapsulated PLGA NPs and the effect of two types of adjuvant including TB10.4 and CpG on immunization, the mice were injected 3 times by using a 0.3-ml insulin syringe (Becton Dickinson, Mountain View, Calif.). Mice were randomly divided into 10 groups (n = 6 in each group) as shown in Table 1. Some groups received one dose of BCG at day 0 (A, B, C, D, E, and G groups) as a prime-boost vaccination. Animals were then immunized with pcDNA3.1/Mtb72F (here abbreviated to DNA) either solution or loaded in NP, DNA loaded in NP with either CpG or TB10.4 at days 7, 14, and 21. The mice received 50 μg of DNA or empty vector (as a control DNA) at each injection. The amount of CpG and TB10.4 were 10 µg and 5 µg, respectively at each injection. Animals immunized with blank-NPs were used as controls. Two weeks after the last immunization, spleens were collected for testing immune responses. All mice were euthanized by cervical dislocation. Spleen samples were harvested under aseptic condition and transferred to the cell lab in the RPMI complete media to prepare the splenocytes as described in the following section.

***Isolation of spleen cells and cytokine production***

The spleen was removed aseptically from all mice in 14 groups. Splenocytes were then isolated and dispersed by RPMI 1640 medium aspiration under safety cabinet. The spleen cells were transferred to a 15-ml conical tube on ice, and washed with fresh media after centrifugation at 500 ×g for 10 min. The cell pellet was resuspended in the RPMI 1640 (Bioidea, Iran) media supplemented with 100 U/ml penicillin and 100 mg/ml streptomycin (Bioidea, Iran) to give a final concentration of 1×10^7^ cells/ml. The cells were incubated on ice until cultured in sterile flat-bottom 24-well plates (Thermoscientific) in RPMI 1640 supplemented with 10% FBS (Bioidea, Iran) and antibiotics. Three hundred microlitres of the cell suspension were added into each well and incubated at 37 ˚C in a humidified CO_2_ incubator with either 10^6^ CFU attenuated *Mycobacterium bovis* BCG (Pasteur institute of Iran) as a stimulator or 3% phytohemeagglutinin (24) as a positive control. The supernatants from three separate wells were collected after 72 hr and pooled and stored at −80 ^°^C until further analysis. 

***IL-4 and IFN-*** ***ɣ*** ***detection***

Interluekin-4 (IL-4) (zellbio; Germany) and interferon-ɣ (IFN-ɣ) (eBioscience;US) were detected in the cell supernatants by commercial Enzyme Linked ImmunoSorbent Assay kits according to the manufacturer’s instructions. 

***Statistical analysis***

Data were analyzed using IBM SPSS (Statistical Package for Social Sciences, version 20.0, SPSS Inc., Chicago, IL) statistics software. The one-way ANOVA test was applied to analyze the difference in cytokine production among different immunized groups. Statistical significance was designated as *P*-value<0.05.

## Results

***Physicochemical properties of PLGA nanoparticles***

DNA with either TB10.4 or CpG was encapsulated in NPs using W/O/W double emulsion method and characterized for size and polydispersity index (PDI). In this study, all experimental setups were performed using bovine serum albumin and empty vector. According to Table 2, the mean diameter of blank NPs was 318.45±2.75 (PDI= 0.375). Either protein-loaded NPs or DNA-loaded NPs were smaller in diameter (mean size=261.45±31.89, PDI=0.305; mean size=181.25±20.15, PDI=0.22, respectively) compared to the blank-NPs. In this research, nucleic acid molecule was sufficiently encapsulated in NPs with EE of 80%. Protein EE was 24% (Table 2).

***Immunological characterization***

To investigate the immune response of our vaccine, we studied Th1- and Th2-associated cytokine production, and antibody titers in immunized mice. 

***Production of cytokines***

**Figure 1:**
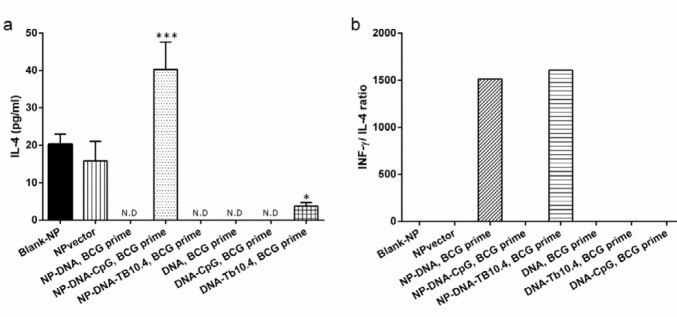
INF-ɣ assay in different immunized mice. The effect of PLGA NP delivery system and BCG priming on the level of INF-ɣ. All data were presented as mean±SD. (*P-value<0.05 compared to negative control). IFN-γ: Interferon gamma, PLGA: Poly (lactic-co-glycolic acid), NP: Nanoparticle, BCG: Bacilli Calmette-Guérin

After splenocytes were harvested from immunized mice, these cells were stimulated with BCG for 72 hr. To evaluate the Th1 activation by our vaccine, the production of INF-ɣ was measured after *in vitro* stimulation of splenocytes from immunized mice with BCG. The supernatants were harvested for ELISA after 72 hr of the stimulation. The amount of INF-ɣ in the mice immunized by NP-encapsulated vaccines (A, B, and G groups) was significantly higher in comparison with the lacking delivery system (C, D, and E groups) (Figure 1). There was no difference of INF-ɣ production between two different vaccination regime (BCG-primed (A, B, and G groups) and No priming (F and I groups)) (Figure 1).

**Figure 2:**
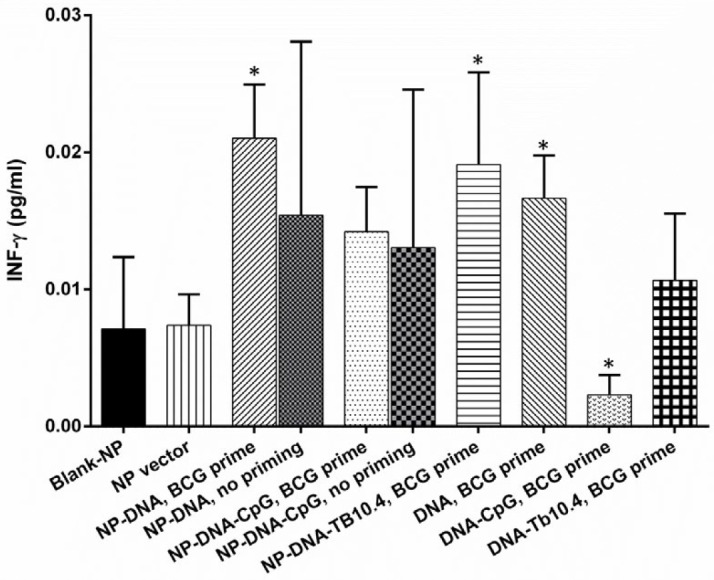
IL-4 assay in different immunized mice. (a) The effect of PLGA NP delivery system on IL-4, (b) the ratio of INF- ɣ to IL-4 after BCG priming and DNA vaccine boost regimen. All data were presented as mean±SD. (*P-value <0.05, ***P-value <0.001 compared to negative control). IL-4: Interleukin, IFN-γ: Interferon gamma, PLGA: Poly (lactic-co-glycolic acid), NP: Nanoparticle, BCG: Bacilli Calmette-Guérin

Th2 responses were evaluated using ELISA measurement of IL-4 secreted by BCG-stimulated spleen cells from immunized mice. The results revealed that immunization of BALB/C mice with blank-NP and NP-control DNA groups as well as NP-DNA-CpG BCG prime induced the production of IL-4 (Figure 2a). 

In order to estimate the Th1/Th2 balance, the INF-ɣ/IL-4 ratio was considered as a simple and reliable indicator according to previous studies (25, 26). A significant high IFN-ɣ/IL-4 ratio implicates a trend toward Th1 bias (27). As shown in Figure 2b, stimulation of the spleen cells from the mice immunized with lacking NP delivery system-vaccine could not induce detectable amount of the INF-ɣ to IL-4 ratio. However, this ratio was higher in mice immunized with NP-DNA-TB10.4-BCG (B group) than that of the mice immunized with NP-DNA-BCG (G group) formulation (Figure 2b), which is demonstrating a strong Th1 response in B group. 

**Table 1:** Study groups and the composition of different vaccines

			Vaccine component					
	Group &Vaccine		pcDNA3.1/Mtb72F	Control DNA (empty vector)	CpG	TB10.4	PLGA NP	BCG priming
**With adjuvant and delivery system**	A	NP-DNA-CpG-BCG	+		+		+	**+**
	B	NP-DNA-TB10.4-BCG	+			+	+	**+**
**No adjuvant no delivery system**	C	DNA-BCG	+					**+**
**No delivery system**	D	DNA-CpG-BCG	+		+			**+**
	E	DNA-TB10.4-BCG	+			+		**+**
**No adjuvant**	F	NP-DNA	+				+	
	G	NP-DNA-BCG	+				+	**+**
	H	NP-control DNA		+			+	
	I	NP-DNA-CpG	+		+		+	
**control**	J	Blank-NP					+	

NPs: nanoparticles; DNA: pcDNA3.1/Mtb72F DNA vaccine; CpG: CpG oligodeoxynucleotides; TB10.4: TB10.4 recombinant protein; BCG: Bacillus Calmette–Guérin vaccine

**Table 2:** Physicochemichal characterization of vector-NPs and protein-NPs by encapsulation efficiency (EE %), size and polydispersity index (PDI) of NPs

Formulation	EE%	Size (28)	PDI
Blank-NPs	-	318.45±2.75	0.375
Protein-NPs	24%	261.45±31.89	0.305
DNA-NPs	80%	181.25±20.15	0.22

NPs: nanoparticles; EE: encapsulation efficiency; PDI: polydispersity index

## Discussion

A challenging area in the field of DNA vaccination is the limited induction of desired immune response (28). Developing a proper NP delivery system (29) and providing an appropriate adjuvant treatment through a prime-boost regimen (30, 31) can enhance the immunogenicity of a plasmid DNA vaccine. In this study, all vaccine formulations were administered either solely or in a BCG-prime boost regimen. The recombinant TB10.4 protein and CpG were investigated as adjuvants in order to induce desired immune responses. 

In this study, the mice were immunized with plasmid DNA vector encoding Mtb72F, a mycobacterium fusion antigen. Our findings showed that this DNA vaccine induced a strong Th1 immunity. It has been reported that Mtb72F that is constructed by linking Mtb32A and Mtb39A can be one of the candidates for the development of TB vaccine (32-34). Both naked DNA and recombinant protein of Mtb72F elicited the immune response and showed protective effect in C57BL/6 mice (32). The use of plasmid DNA encoding fusion mycobacterial antigens is a promising approach to increase the potency of DNA vaccine against *M. tuberculosis (*35*)*. The studies with a DNA plasmid vaccine encoding Ag85A-Tb10.4 (36) and Mtb32C-HBHA (30) fusion antigen showed significantly increased levels of IFN-γ and IL-12 production. Inconsistently, the DNA plasmid expressing Ag85B-ESAT-6 fusion protein could not relatively improve protective efficacy above than that of the achievement by each individual constructs (37), while the Ag85B-ESAT-6 fusion protein has been studied in Phase II clinical trials.

Our experiments demonstrated that immunization of BALB/c mice with NP-DNA elicited higher INF-ɣ (Figure 1), the hallmark of Th1cytokine, and greater INF-ɣ/IL-4 ratio compared to the naked DNA vaccine (Figure 2b). These values correlate favorably with Lima *et al.* and Bivas-Benita *et al.* (38, 39), which further support the idea that PLGA encapsulation could induced very strong Th1 responses. 

PLGA based NP is one of the biodegradable and FDA approved approach in drug and vaccine delivery system (40, 41). It has now been suggested that encapsulation of DNA or subunit vaccines in particles may enhance their immunogenicity by controlled release of vaccine, improvement of their stability, increasing their dendritic cells (DCs) uptake, and promoting cross-presentation and cytotoxic T lymphocyte response (42). The particle size of PLGA NPs is the crucial characteristics affecting their biodistribution and subsequently the interaction of NPs with immune cells. Particles in the range of 20-200 nm efficiently induce cellular immune responses through their endocytosis or pinocytosis by DCs, whereas the larger particles in the range of 0.5-5 µm mainly generate humoral responses via phagocytosis or macropinocytosis of the particles (42). Our study provides an effective double emulsion protocol resulting in fine NPs smaller than 250 nm (Table 2). 

Several researchers have used the prime-boost strategy to enhance the immunogenicity of DNA vaccine (35). Interestingly, priming with BCG and boosting with NP-DNA or NP-DNA-TB10.4 showed a high level of cytokine production from spleen cells. This finding is in line with previous results (43-46). Administration of BCG and DNA vaccines encoding RV0577 (43), and co-expressed CFP10, ESAT6, Ag85A and Ag85B (44) resulted in prolonged Th1 immune response and increased production of cytokine including IFN-ɣ. In the first phase I study, it has been reported that recombinant modified vaccinia virus Ankara (MVA) expressing antigen 85A (MVA85A) could induce high level of antigen specific T-cells compared to single BCG vaccination (45). PLGA:DDA hybrid NP encapsulated with HspX/EsxS multistage subunit and MPLA adjuvant induced the highest INF-γ and IgG2a and IgG1 level and has a great potential for promoting BCG efficacy as a BCG prime-boost (46). 

To identify the suitable adjuvant for DNA vaccine, the soluble and encapsulated form of CpG and TB10.4 recombinant protein were administered. As shown in Figure 1a, TB10.4 induced significantly higher IFN-ɣ compared to CpG. The TB10.4 protein has been previously shown to stimulate strong CD4^+^ and CD8^+^ T-cell responses and produce Th1 cytokines (8). It is interesting to note that the formulations that induce Th1 responses are perfectly desirable for efficient vaccination (32). Therefore, these results offer the valuable adjuvant, TB10.4, for DNA vaccine against TB. 

## Conclusion

Our results demonstrate that the administration of PLGA NPs encapsulated with Mtb72F plasmid and purified TB10.4 recombinant protein can induce Th1 cytokine production in a BCG-prime boost strategy. In fact, this formulation has successfully enhanced the efficacy of BCG vaccine in an animal model. The current study is the first step in investigating the immunogenicity phase, toward development of NP-based DNA vaccine to protect mice against TB. Therefore, additional experiments will be needed including *M. tuberculosis* challenge study to show the complete protective efficacy of these formulations against TB infection.
